# *N*′-(3-Bromo-5-chloro-2-hydroxy­benzyl­idene)-2-methoxy­benzohydrazide

**DOI:** 10.1107/S1600536809010198

**Published:** 2009-03-25

**Authors:** Jin-Long Hou

**Affiliations:** a College of Chemistry and Chemical Engineering, Qiqihar University, Qiqihar 161006, People’s Republic of China

## Abstract

The title compound, C_15_H_12_BrClN_2_O_3_, was obtained by the condensation reaction between 3-bromo-5-chloro-2-hydroxy­benzaldehyde and 2-methoxy­benzohydrazide. The mol­ecule is essentially planar, with a dihedral angle between the two benzene rings of 4.7 (2)°, and displays an *E* configuration about the C=N double bond. The mol­ecular conformation is stabilized by intramolecular O—H⋯N and N—H⋯O hydrogen bonds. In the crystal structure, mol­ecules are linked into zigzag chains running parallel to the *c* axis by inter­molecular C—H⋯O hydrogen bonds. The chains are further connected through aromatic π–π stacking inter­actions with centroid–centroid distances of 3.583 (4) Å.

## Related literature

For the biological properties of hydrazone compounds, see: Cukurovali *et al.* (2006 ▶); Karthikeyan *et al.* (2006 ▶); Kucukguzel *et al.* (2006 ▶). For the crystal structures of related hydrazone compounds, see: Mohd Lair *et al.* (2009 ▶); Fun *et al.* (2008 ▶); Zhang *et al.* (2009 ▶); Khaledi *et al.* (2008 ▶). For bond-length data, see: Allen *et al.* (1987 ▶).
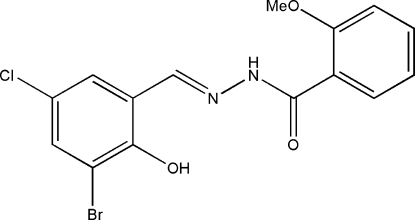


## Experimental

### 

#### Crystal data

C_15_H_12_BrClN_2_O_3_
*M* *_r_* = 383.63
Monoclinic, 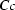

*a* = 10.883 (1) Å
*b* = 12.863 (2) Å
*c* = 10.950 (1) Å
β = 96.027 (3)°
*V* = 1524.4 (3) Å^3^
*Z* = 4
Mo *K*α radiation
μ = 2.89 mm^−1^
*T* = 298 K
0.12 × 0.12 × 0.10 mm

#### Data collection

Bruker SMART 1000 CCD area-detector diffractometer
Absorption correction: multi-scan (*SADABS*; Sheldrick, 1996 ▶) *T*_min_ = 0.709, *T*_max_ = 0.746
4397 measured reflections
2323 independent reflections
1906 reflections with *I* > 2σ(*I*)
*R*_int_ = 0.028

#### Refinement

*R*[*F* ^2^ > 2σ(*F* ^2^)] = 0.033
*wR*(*F* ^2^) = 0.062
*S* = 1.02
2323 reflections
205 parameters
3 restraints
H atoms treated by a mixture of independent and constrained refinement
Δρ_max_ = 0.32 e Å^−3^
Δρ_min_ = −0.28 e Å^−3^
Absolute structure: Flack (1983 ▶), 671 Friedel pairs
Flack parameter: 0.068 (12)

### 

Data collection: *SMART* (Bruker, 1998 ▶); cell refinement: *SAINT* (Bruker, 1998 ▶); data reduction: *SAINT*; program(s) used to solve structure: *SHELXS97* (Sheldrick, 2008 ▶); program(s) used to refine structure: *SHELXL97* (Sheldrick, 2008 ▶); molecular graphics: *SHELXTL* (Sheldrick, 2008 ▶); software used to prepare material for publication: *SHELXTL*.

## Supplementary Material

Crystal structure: contains datablocks global, I. DOI: 10.1107/S1600536809010198/rz2301sup1.cif

Structure factors: contains datablocks I. DOI: 10.1107/S1600536809010198/rz2301Isup2.hkl

Additional supplementary materials:  crystallographic information; 3D view; checkCIF report

## Figures and Tables

**Table 1:** Hydrogen-bond geometry (Å, °)

*D*—H⋯*A*	*D*—H	H⋯*A*	*D*⋯*A*	*D*—H⋯*A*
O1—H1⋯N1	0.82	1.83	2.549 (4)	146
N2—H2⋯O3	0.895 (10)	1.92 (3)	2.623 (4)	134 (4)
C6—H6⋯O2^i^	0.93	2.45	3.315 (6)	154

Symmetry code: (i) 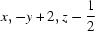.

**Fig. 1.**
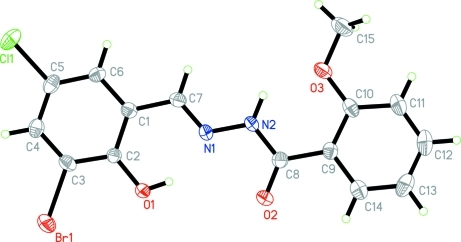
Molecular structure with displacement ellipsoids drawn at 30% probability for non-H atoms.

## supplementary crystallographic information

### Comment

Hydrazones derived from the condensation reactions of hydrazides with aldehydes show excellent biological properties (Cukurovali *et al.*, 2006; Karthikeyan *et al.*, 2006; Kucukguzel *et al.*, 2006). In the last two years, several hydrazone compounds have been structurally characterized (Mohd Lair *et al.*, 2009; Fun *et al.*, 2008; Zhang *et al.*, 2009; Khaledi *et al.*, 2008). In this paper, the synthesis and crystal structure of the title compound, derived from the condensation reaction of 3-bromo-5-chloro-2-hydroxybenzaldehyde and 2-methoxybenzohydrazide, is reported.

The molecular structure of the title compound is shown in Fig. 1. The molecule is essentially planar (mean deviation 0.010 (4) Å) and displays an *E* configuration about the C═N double bond. All bond lengths are within normal ranges (Allen *et al.*, 1987). The molecular conformation is stabilized by intramolecular O—H···N and N—H···O hydrogen bonds (Table 1). In the crystal structure, the molecules are linked into zig-zag chains running parallel to the *c* axis by intermolecular C—H···O hydrogen bonds. The chains are further connected by aromatic π-π stacking interactions: Cp1···Cp2^i^ = 3.583 (4) Å, perpendicular interplanar distance = 3.430 (4) Å, Cp1···Cp2^i^ offset = 1.037 (3) Å [Cp1 and Cp2 are the centroids of the C9–C14 and C1–C6 armatic rings, respectively. Symmetry code: (i) -1/2+x, -1/2+y, z].

### Experimental

3-Bromo-5-chloro-2-hydroxybenzaldehyde (1.0 mmol, 235.5 mg) and 2-methoxybenzohydrazide (1.0 mmol, 166.2 mg) were mixed and refluxed with stirring for two hours. Yellow single crystals were formed after slow evaporation of the solution in air for a week.

### Refinement

H2 was located in a difference Fourier map and refined isotropically, with the N–H distance restrained to 0.90 (1) Å, and with the *U*_iso_(H) value fixed at 0.08 Å^2^. All other H atoms were placed in idealized positions and constrained to ride on their parent atoms, with C–H = 0.93–0.96 Å, O–H = 0.82 Å, and with *U*_iso_(H) set at 1.2*U*_eq_(C) or 1.5*U*_eq_(O).

### Crystal data

**Table:**

C_15_H_12_BrClN_2_O_3_	*F*(000) = 768
*M**_r_* = 383.63	*D*_x_ = 1.672 Mg m^−^^3^
Monoclinic, *C**c*	Mo *K*α radiation, λ = 0.71073 Å
Hall symbol: C -2yc	Cell parameters from 1920 reflections
*a* = 10.883 (1) Å	θ = 2.4–25.0°
*b* = 12.863 (2) Å	µ = 2.89 mm^−^^1^
*c* = 10.950 (1) Å	*T* = 298 K
β = 96.027 (3)°	Block, yellow
*V* = 1524.4 (3) Å^3^	0.12 × 0.12 × 0.10 mm
*Z* = 4	

### Data collection

**Table:**

Bruker SMART 1000 CCD area-detector diffractometer	2323 independent reflections
Radiation source: fine-focus sealed tube	1906 reflections with *I* > 2σ(*I*)
graphite	*R*_int_ = 0.028
ω scans	θ_max_ = 27.0°, θ_min_ = 2.5°
Absorption correction: multi-scan (*SADABS*; Sheldrick, 1996)	*h* = −13→12
*T*_min_ = 0.709, *T*_max_ = 0.746	*k* = −16→16
4397 measured reflections	*l* = −13→13

### Refinement

**Table:**

Refinement on *F*^2^	Secondary atom site location: difference Fourier map
Least-squares matrix: full	Hydrogen site location: inferred from neighbouring sites
*R*[*F*^2^ > 2σ(*F*^2^)] = 0.033	H atoms treated by a mixture of independent and constrained refinement
*wR*(*F*^2^) = 0.062	*w* = 1/[σ^2^(*F*_o_^2^) + (0.0041*P*)^2^] where *P* = (*F*_o_^2^ + 2*F*_c_^2^)/3
*S* = 1.02	(Δ/σ)_max_ < 0.001
2323 reflections	Δρ_max_ = 0.32 e Å^−^^3^
205 parameters	Δρ_min_ = −0.28 e Å^−^^3^
3 restraints	Absolute structure: Flack (1983), 671 Friedel pairs
Primary atom site location: structure-invariant direct methods	Flack parameter: 0.068 (12)

### Special details

**Table:**

Geometry. All e.s.d.'s (except the e.s.d. in the dihedral angle between two l.s. planes) are estimated using the full covariance matrix. The cell e.s.d.'s are taken into account individually in the estimation of e.s.d.'s in distances, angles and torsion angles; correlations between e.s.d.'s in cell parameters are only used when they are defined by crystal symmetry. An approximate (isotropic) treatment of cell e.s.d.'s is used for estimating e.s.d.'s involving l.s. planes.
Refinement. Refinement of *F*^2^ against ALL reflections. The weighted *R*-factor *wR* and goodness of fit *S* are based on *F*^2^, conventional *R*-factors *R* are based on *F*, with *F* set to zero for negative *F*^2^. The threshold expression of *F*^2^ > σ(*F*^2^) is used only for calculating *R*-factors(gt) *etc*. and is not relevant to the choice of reflections for refinement. *R*-factors based on *F*^2^ are statistically about twice as large as those based on *F*, and *R*- factors based on ALL data will be even larger.

### Fractional atomic coordinates and isotropic or equivalent isotropic displacement parameters (Å^2^)

**Table:**

	*x*	*y*	*z*	*U*_iso_*/*U*_eq_	
Br1	0.87674 (5)	1.27290 (3)	0.55418 (4)	0.05914 (15)	
Cl1	0.79439 (11)	1.45929 (8)	0.10011 (11)	0.0630 (3)	
O3	0.5131 (3)	0.7894 (2)	−0.0042 (3)	0.0538 (9)	
O2	0.6516 (3)	0.8295 (2)	0.3590 (3)	0.0546 (9)	
C8	0.6188 (3)	0.8209 (3)	0.2494 (4)	0.0396 (9)	
N2	0.6245 (3)	0.9033 (2)	0.1728 (3)	0.0418 (8)	
N1	0.6674 (3)	0.9949 (2)	0.2236 (3)	0.0392 (7)	
O1	0.7599 (3)	1.09258 (19)	0.4140 (2)	0.0444 (6)	
H1	0.7332	1.0421	0.3738	0.067*	
C7	0.6737 (3)	1.0742 (3)	0.1564 (4)	0.0415 (9)	
H7	0.6477	1.0713	0.0728	0.050*	
C2	0.7652 (3)	1.1748 (3)	0.3396 (3)	0.0344 (8)	
C6	0.7306 (4)	1.2597 (3)	0.1403 (5)	0.0390 (11)	
H6	0.7025	1.2577	0.0571	0.047*	
C9	0.5699 (4)	0.7207 (3)	0.1948 (4)	0.0383 (9)	
C1	0.7228 (3)	1.1710 (3)	0.2134 (3)	0.0370 (9)	
C5	0.7799 (3)	1.3492 (3)	0.1920 (4)	0.0447 (10)	
C3	0.8145 (3)	1.2676 (3)	0.3864 (4)	0.0420 (9)	
C4	0.8216 (4)	1.3547 (3)	0.3147 (4)	0.0444 (10)	
H4	0.8541	1.4163	0.3487	0.053*	
C10	0.5176 (3)	0.7058 (3)	0.0733 (4)	0.0433 (10)	
C11	0.4706 (4)	0.6082 (3)	0.0378 (4)	0.0528 (11)	
H11	0.4330	0.5987	−0.0417	0.063*	
C15	0.4532 (5)	0.7794 (4)	−0.1255 (4)	0.0696 (15)	
H15A	0.3678	0.7624	−0.1219	0.104*	
H15B	0.4922	0.7252	−0.1678	0.104*	
H15C	0.4593	0.8438	−0.1687	0.104*	
C14	0.5754 (4)	0.6344 (4)	0.2755 (4)	0.0486 (12)	
H14	0.6094	0.6422	0.3565	0.058*	
C13	0.5317 (4)	0.5407 (4)	0.2359 (5)	0.0645 (13)	
H13	0.5374	0.4845	0.2896	0.077*	
C12	0.4787 (4)	0.5271 (4)	0.1169 (5)	0.0661 (13)	
H12	0.4485	0.4622	0.0911	0.079*	
H2	0.597 (4)	0.897 (4)	0.0932 (14)	0.080*	

### Atomic displacement parameters (Å^2^)

**Table:**

	*U*^11^	*U*^22^	*U*^33^	*U*^12^	*U*^13^	*U*^23^
Br1	0.0772 (3)	0.0527 (2)	0.0443 (2)	−0.0058 (3)	−0.00881 (19)	−0.0139 (3)
Cl1	0.0775 (8)	0.0393 (6)	0.0743 (8)	0.0053 (5)	0.0180 (6)	0.0199 (6)
O3	0.066 (2)	0.059 (2)	0.0339 (18)	−0.0160 (16)	−0.0040 (16)	−0.0048 (16)
O2	0.080 (2)	0.049 (2)	0.0320 (18)	−0.0123 (16)	−0.0084 (16)	−0.0009 (14)
C8	0.041 (2)	0.038 (2)	0.040 (2)	0.0012 (17)	0.0042 (18)	−0.0006 (19)
N2	0.058 (2)	0.0367 (19)	0.0294 (17)	−0.0104 (15)	−0.0027 (15)	−0.0052 (15)
N1	0.0491 (18)	0.0338 (18)	0.0338 (17)	−0.0084 (14)	0.0003 (14)	−0.0040 (14)
O1	0.0669 (17)	0.0337 (16)	0.0304 (14)	−0.0053 (13)	−0.0056 (13)	−0.0023 (12)
C7	0.049 (2)	0.044 (2)	0.031 (2)	0.0010 (18)	−0.0001 (17)	−0.0006 (18)
C2	0.041 (2)	0.028 (2)	0.034 (2)	0.0049 (15)	0.0012 (17)	−0.0020 (16)
C6	0.042 (2)	0.037 (3)	0.038 (3)	0.0041 (18)	0.0027 (19)	0.0063 (18)
C9	0.040 (2)	0.036 (2)	0.041 (2)	−0.0012 (16)	0.0115 (18)	−0.0041 (18)
C1	0.0378 (19)	0.037 (2)	0.036 (2)	0.0046 (17)	0.0020 (17)	−0.0033 (17)
C5	0.045 (2)	0.031 (2)	0.059 (3)	0.0068 (18)	0.013 (2)	0.006 (2)
C3	0.045 (2)	0.045 (2)	0.035 (2)	0.0050 (18)	−0.0012 (18)	−0.0095 (19)
C4	0.050 (2)	0.029 (2)	0.055 (3)	0.0033 (16)	0.008 (2)	−0.0042 (18)
C10	0.038 (2)	0.050 (3)	0.043 (2)	−0.0062 (17)	0.0095 (18)	−0.0154 (19)
C11	0.053 (3)	0.052 (3)	0.055 (3)	−0.015 (2)	0.012 (2)	−0.018 (2)
C15	0.073 (3)	0.089 (4)	0.044 (3)	−0.023 (3)	−0.005 (2)	−0.011 (3)
C14	0.045 (2)	0.052 (3)	0.049 (3)	−0.006 (2)	0.008 (2)	−0.010 (2)
C13	0.071 (3)	0.043 (3)	0.083 (4)	−0.004 (2)	0.025 (3)	0.012 (3)
C12	0.067 (3)	0.044 (3)	0.090 (4)	−0.014 (2)	0.020 (3)	−0.018 (3)

### Geometric parameters (Å, °)

**Table:**

Br1—C3	1.891 (4)	C6—H6	0.9300
Cl1—C5	1.754 (4)	C9—C10	1.404 (6)
O3—C10	1.367 (5)	C9—C14	1.417 (6)
O3—C15	1.423 (5)	C5—C4	1.374 (6)
O2—C8	1.221 (4)	C3—C4	1.375 (6)
C8—N2	1.357 (5)	C4—H4	0.9300
C8—C9	1.494 (5)	C10—C11	1.396 (5)
N2—N1	1.364 (4)	C11—C12	1.353 (7)
N2—H2	0.895 (10)	C11—H11	0.9300
N1—C7	1.265 (4)	C15—H15A	0.9600
O1—C2	1.341 (4)	C15—H15B	0.9600
O1—H1	0.8200	C15—H15C	0.9600
C7—C1	1.468 (5)	C14—C13	1.350 (6)
C7—H7	0.9300	C14—H14	0.9300
C2—C3	1.384 (5)	C13—C12	1.379 (7)
C2—C1	1.411 (5)	C13—H13	0.9300
C6—C5	1.367 (6)	C12—H12	0.9300
C6—C1	1.402 (6)		
			
C10—O3—C15	119.4 (4)	C4—C3—C2	122.2 (4)
O2—C8—N2	120.6 (4)	C4—C3—Br1	119.3 (3)
O2—C8—C9	121.8 (4)	C2—C3—Br1	118.5 (3)
N2—C8—C9	117.6 (4)	C5—C4—C3	118.9 (4)
C8—N2—N1	117.3 (3)	C5—C4—H4	120.5
C8—N2—H2	120 (3)	C3—C4—H4	120.5
N1—N2—H2	123 (3)	O3—C10—C11	123.1 (4)
C7—N1—N2	119.8 (3)	O3—C10—C9	117.7 (3)
C2—O1—H1	109.5	C11—C10—C9	119.1 (4)
N1—C7—C1	118.4 (3)	C12—C11—C10	121.2 (4)
N1—C7—H7	120.8	C12—C11—H11	119.4
C1—C7—H7	120.8	C10—C11—H11	119.4
O1—C2—C3	119.6 (3)	O3—C15—H15A	109.5
O1—C2—C1	122.5 (3)	O3—C15—H15B	109.5
C3—C2—C1	117.9 (3)	H15A—C15—H15B	109.5
C5—C6—C1	119.6 (4)	O3—C15—H15C	109.5
C5—C6—H6	120.2	H15A—C15—H15C	109.5
C1—C6—H6	120.2	H15B—C15—H15C	109.5
C10—C9—C14	118.1 (4)	C13—C14—C9	120.6 (4)
C10—C9—C8	126.1 (4)	C13—C14—H14	119.7
C14—C9—C8	115.9 (4)	C9—C14—H14	119.7
C6—C1—C2	119.8 (4)	C14—C13—C12	121.0 (5)
C6—C1—C7	119.3 (4)	C14—C13—H13	119.5
C2—C1—C7	120.9 (3)	C12—C13—H13	119.5
C6—C5—C4	121.5 (4)	C11—C12—C13	119.9 (4)
C6—C5—Cl1	119.8 (4)	C11—C12—H12	120.0
C4—C5—Cl1	118.6 (3)	C13—C12—H12	120.0

### Hydrogen-bond geometry (Å, °)

**Table:**

*D*—H···*A*	*D*—H	H···*A*	*D*···*A*	*D*—H···*A*
O1—H1···N1	0.82	1.83	2.549 (4)	146
N2—H2···O3	0.90 (1)	1.92 (3)	2.623 (4)	134 (4)
C6—H6···O2^i^	0.93	2.45	3.315 (6)	154

Symmetry codes: (i) *x*, −*y*+2, *z*−1/2.
